# p53 and the Viral Connection: Back into the Future ‡

**DOI:** 10.3390/cancers10060178

**Published:** 2018-06-04

**Authors:** Ronit Aloni-Grinstein, Meital Charni-Natan, Hilla Solomon, Varda Rotter

**Affiliations:** 1 Department of Molecular Cell Biology, Weizmann Institute of Science, 76100 Rehovot, Israel; ronitag@iidr.gov.il (R.A.-G.); meital.charni@weizmann.ac.il (M.C.-N.); hilla.besserglick@weizmann.ac.il (H.S.); 2 Department of Biochemistry and Molecular Genetics, Israel Institute for Biological Research, Box 19, 74100 Ness-Ziona, Israel

**Keywords:** virus, p53, cancer, SV40, papilloma, zika, influenza, HIV-1, HSV-1, vaccinia

## Abstract

The discovery of the tumor suppressor p53, through its interactions with proteins of tumor-promoting viruses, paved the way to the understanding of p53 roles in tumor virology. Over the years, accumulating data suggest that WTp53 is involved in the viral life cycle of non-tumor-promoting viruses as well. These include the influenza virus, smallpox and vaccinia viruses, the Zika virus, West Nile virus, Japanese encephalitis virus, Human Immunodeficiency Virus Type 1, Human herpes simplex virus-1, and more. Viruses have learned to manipulate WTp53 through different strategies to improve their replication and spreading in a stage-specific, bidirectional way. While some viruses require active WTp53 for efficient viral replication, others require reduction/inhibition of WTp53 activity. A better understanding of WTp53 functionality in viral life may offer new future clinical approaches, based on WTp53 manipulation, for viral infections.

## 1. Introduction

Almost 40 years ago, while studying tumor induction by small DNA tumor viruses, several groups have “bumped” into a nonviral protein with an apparent molecular mass of 53KDa, which co-immunoprecipitated with Simian Vacuolating Virus 40 (SV40) large T-antigen protein [1,2,3,4,5]. This host protein was also identified in most of the retrovirus Abelson Murine Leukemia Virus (A-MuLV) infected transformed cells [6]. The question of whether p53 is an oncogene or a tumor suppressor was for many years an enigma. Finally, it was understood that WTp53 is a tumor suppressor gene, which was crowned as the “guardian of the genome” [7], while the mutant p53 is a bona fide oncogene [8].

Indeed, WTp53 is a well-known tumor suppressor protein, which is activated by varied stress signals. When activated, WTp53 regulates various cellular pathways that determine the cell fate, such as cell cycle arrest, differentiation, senescence, and apoptosis [7,9,10]. As viral infection evokes cellular stress, it is not surprising that infected cells harbor stabilized activated WTp53 [11]. Consequently, to gain successful replication and spreading, viruses use different strategies to handle their host cells and manipulate WTp53’s guardian role. Different viral families have evolved protein binding motifs and other mechanisms to either hijack or interfere with WTp53 functions. For example, in order to spread, some viruses cause WTp53-mediated cell death of the host cell by different mechanisms such as cell lysis and various types of programmed cell death (e.g., apoptosis). On the other hand, some viruses can cause cells to proliferate by attenuating WTp53 function and by that may seed the onset of cancer development [12]. Of note, it is well established that in addition to the interaction of p53 with viruses, cancer is also associated with the direct mutation of the p53 gene. Indeed, p53 is mutated in more than 50% of human tumors. Most of the common p53 mutations gained an oncogenic function rather than just being a dysfunctional tumor suppressor gene [8,13].

In this review, we discuss the different viral strategies used to modulate the p53 defensive responses.

## 2. Tumor-Promoting Viruses

### 2.1. SV40 and Adenoviruses

The small DNA viruses, Adenoviruses and SV40, utilize the DNA replication machinery of the host cell by elegant and diverse strategies. In the host cell, transcription regulation of cell cycle genes is achieved by the binding of the Retinoblastoma protein (Rb) to the transcription factor E2F. Apparently, both Adenoviruses and SV40 are able to manipulate the host cell by the binding of their viral proteins, E1A and SV40 T-antigen, to the Rb, respectively [14,15,16,17,18,19]. This binding releases Rb from E2F, leading to E2F transcriptional activation that mediates S phase entrance. p53, as the “guardian of the genome”, senses this aberrant S phase movement and acts to induce apoptosis. However, to eliminate p53 activity, the viral T-antigen or the adenovirus E1B proteins bind p53 and by that inactivate it [20], allowing the progression into the S phase [9].

Another association between SV40 and p53 is noticed on the transcription level. Studies suggested that T-antigen uses “DNA mimicry” [9]; T-antigen contains p53 elements that are able to bind to p53 and by that prevent p53 transcriptional regulation, leading to transformation of the host cell [21]. Indeed, all examined T-antigen mutants that were no longer able to bind p53 lost their ability to transform the infected cells [22]. Moreover, this association of p53 with SV40 not only attenuates p53 normal functions, but also exerts a “helper” function for SV40 prosperity. This helper function is provided by WTp53 through its TAD1 domain, which bridges the interactions of p53 with various transcriptional promoting proteins such as histone acetyltransferase p300 and CBP, thus providing SV40 with additional transcriptional potential [23].

Drayman et al. provided another view on the relationship between p53 and SV40. Interestingly, immediately after SV40 infection, activated p53 is noticed only in cells that do not express SV40 viral proteins, suggesting a p53-dependent decision between abortive and productive infection. These p53 host defense mechanisms did not rely on apoptosis, cell cycle arrest, or induction of interferon-stimulated genes, but rather on binding competition between p53 and Sp1, a host protein, which is essential for SV40 assembly [24,25].

### 2.2. Papilloma

The human papilloma viruses (HPVs) are double-stranded DNA viruses with over 60 identified types, which are divided by their oncogenic capacity into two groups, high-risk and low-risk HPVs. While low-risk HPVs are associated with benign hyperplasia, high-risk HPVs contain 12 known virus types that are associated with anogenital carcinomas. Although the majority of cervical cancer cases are caused by the high-risk HPV-16 and HPV-18 infections, other cancer types are also associated with high-risk HPVs such as head and neck, mouth, vulva, vagina, penis, and anus cancers [26,27]. In order to induce viral replication in the infected cells, the HPV takes advantage of its viral proteins E6 and E7 that inhibit the p53 and pRb tumor suppressor proteins activity, respectively. Hence, the HPV interferes with the normal host cell cycle and leads to malignant transformation [28,29]. The viral E6 protein abuses the host protein-degradation machinery by interactions with its E3 ubiquitin ligase, E6AP, which in turn directly mediates the degradation of p53 by 26S proteasome, leading to low levels of p53 in the infected cells. Accordingly, E6 proteins of low-risk HPV types, such as HPV-6 and HPV-11, are incapable of degrading p53 efficiently [30,31,32], thus leading to a benign phenotype.

In addition, recent accumulated data have shown that not only E6 but also the viral protein E7 are able to affect p53 function by their binding to the DREAM complex. p53 regulates the DREAM complex in order to inhibit the expression of plethora of genes. It was found that E7 is able to associate directly with DREAM components p107 and p130, leading to disruption in p53 regulation [33,34]. Taken together, it seems that both the E6 and E7 proteins of HPV interfere with p53 regulation and cause changes in the host cell cycle, resulting in unregulated cell divisions and emission from apoptosis.

## 3. Nononcogenic Viruses

### 3.1. Poxviruses

The smallpox and vaccinia viruses, which are members of the poxviruses family, have a large DNA of 191 kb [35], thus the replication of their viral DNA in the host cell is likely to be a major burden. The B1R kinase is an early viral gene required for vaccinia virus DNA synthesis and replication. B1R was found to hyperphosphorylate p53 in several residues in the N-terminal transactivation domain, including Ser15 and Thr18, in an MDM2-dependent manner, leading to an increase in p53 ubiquitination and degradation. Moreover, the presence of B1R significantly reduced the acetylation of p53 by p300, reducing p53 stability [36]. This mechanism of p53 downregulation was suggested to allow DNA synthesis of poxviruses, such as observed in cells infected with vaccinia virus [37]. Another poxvirus that uses this mechanism is the Tanapoxvirus (TPV), a member of the *Yatapoxvirus* genus [38]. Infection in humans results in a febrile illness, characterized by the formation of a few small, papular lesions that ulcerate but heal rapidly within a few weeks. The open reading frame of the TPV142R shares significant amino acid homology to the B1R kinase and is also capable of phosphorylating p53 and reducing its expression [39]. Accordingly, by mediating p53 destabilization, the B1R and TPV142R proteins allow the vaccinia and TPV viruses to proliferate and prosper inside infected cells.

### 3.2. Flavivirus

The Zika virus (ZIKV) is a mosquito-borne, single-stranded, positive-sense RNA virus belonging to the genus *Flavivirus* in the family *Flaviviridae*, originally identified in Uganda in 1947 [40] and which has spread in Latin America, mainly in Brazil [41]. ZIKV infection in pregnant women was suggested to be associated with the increasing incident of congenital microcephaly (CM) observed since 2015 [41,42,43]. ZIKV infection, as other risk factors including rare genetic disorders or environmental factors such as hypoxia, drugs, or various pathogens, affect the normal neural progenitors expansion, differentiation, and the survival of their progeny. This in turn may lead to reduction in the final number of brain cells and to CM [44]. The mechanisms underlying these biological processes are still obscure. In an attempt to reveal the key factors mediating this phenotype, the gene expression profile of induced pluripotent stem cell derived from human neural progenitors cells (hNPC) infected with ZIKV was compared with the profiles of developing neural tissues obtained from three severe genetic microcephaly models. The study revealed that a signature of p53 regulation is common to ZIKV and to the microcephaly models. This signature consists of antiproliferative and pro-apoptotic p53-related responses. Moreover, it was shown that ZIKV-infected hNPCs are characterized by genotoxic stress, p53 activation, and apoptotic cell death [45]. In another study, a database of 248 ZIKV-related proteins in the human genome and 221 microcephaly-associated human proteins was used to predict their shared molecular mechanisms. Interestingly, p53 was found to possess a central role in the genetic regulatory network of apoptosis and cell death pathways in both ZIKV infection and microcephaly. This reported apoptosis of neural cells is most likely the core for neuronal defects and CM caused upon infection with ZIKV. The suggested mechanism underlying this p53 activation is the binding of the ZIKV capsid protein (ZCP) to p53 E3 ligase, MDM2, thus preventing the formation of the MDM2-p53 complex. In that manner, the ZIKV leads to elevated p53 protein levels and its activation, triggering the apoptosis of neural cells [46].

The West Nile virus, which may cause different severe morbidities and even death [47], is another member of the Flaviridae family which can also elevate p53 levels during its infection process. The West Nile viral capsid protein interacts with MDM2 and sends it to the nucleolus, thus preventing its binding to p53 and leading to p53 stabilization. The stabilized p53 activates Bax protein that in turn mediates apoptosis [48].

### 3.3. Influenza A Virus

Influenza A virus (IAV) is a member of the *Orthomyxovirdae* family of RNA viruses and the primary cause of respiratory tract infections [49]. IAV is a cytolytic virus that induces apoptosis, presumably to improve the efficiency of its replication [50,51]. Indeed, the accumulation and activation of p53 in IAV-infected cells is essential for apoptosis [52,53]. To obtain p53 stabilization, the viral nucleoprotein (NP) impairs the interaction between p53 and MDM2, leading to p53 stability and transcriptional activity [54]. In addition, others reported that NP interacts with the host protein RING finger protein 43 (RNF43), an E3 ubiquitin ligase, in order to modulate p53 ubiquitination levels, leading to p53 stabilization and enhanced apoptosis [55]. Both in vitro and in vivo experiments show correlation between p53 absence and survival of IAV infected cells. For example, reduction of p53 activity in cell culture inhibited IAV-induced cell death; in addition, mouse embryo fibroblasts isolated from p53 knockout mice, infected with IAV, exhibited augmented levels of survival in comparison to wild-type p53 mouse embryo fibroblasts [52]. It seems that IAV hijacks p53 by causing its activation, leading to enhanced apoptosis and thus IAV viral replication. However, ample data suggest that p53 also acts as a host antiviral factor that enhances innate and adaptive immune responses in order to battle the infection. For example, IAV infection of p53-deficient mice resulted in increased mortality, severe weight loss, and increased viral load in their lungs compared with WT p53 counterparts. Indeed, a comparative analysis of the global expression profiles of IAV-infected p53K/O and p53WT mice suggested an impaired interferon-mediated immune response against IAV infection in the absence of p53. Moreover, a comparison of cytokine and chemokine expression levels between IAV-infected p53K/O and p53WT mice implies a dysregulated cytokine and pro-inflammatory chemokine response in the absence of p53 [56]. In agreement with the latter study, knockdown of p53 expression by RNAi enhanced IAV replication and reduced the expression of antiviral type I interferon-stimulated genes (ISGs), such as IRF7, IRF9, ISG15, ISG20, GBP1, RIG1, and OAS1 [57]. Another study showed that p53 serves as a host antiviral factor that enhances innate and adaptive immune responses to IAV. Indeed, lungs of WT p53 mice that were infected with IAV exhibited lower levels of IAV viral load in comparison to lungs of p53K/O mice. Interestingly, the massive leukocyte infiltration into p53K/O mice lungs was observed only in a later phase of infection and was accompanied by a severe necrosis of the bronchial epithelium [58]. Furthermore, p53 absence was associated with defective IAV-specific T-cell immunity. Wang et al. showed that upon influenza virus infection, p53 is activated, leading to elevation of its target gene, endoplasmic reticulum aminopeptidase 1 (ERAP1), which is a regulator of the Major Histocompatibility Complex I (MHCI) expression. Appropriate regulation of MHC expression is important for protection against viral infection [59]. These data propose a role for p53 in the Cytotoxic T lymphocytes MHC1-dependent response [60]. Taken together, these findings suggest that the absence of p53 leads to delayed innate response, which impairs the ability to clear the virus from the lungs. This may cause severe IAV-induced morbidity observed in the p53K/O mice. All in all, it seems that IVA induces p53 accumulation in a biphasic pattern, first at the beginning phase of infection, immediately after the virus absorption, leading to an innate response, and at the middle-late phase of infection at the apoptotic phase [53].

### 3.4. The Human Immunodeficiency Virus Type 1

The human immunodeficiency virus type 1 (HIV-1) is a lentivirus (a subgroup of retrovirus) which causes a progressive loss in CD4 lymphocyte numbers and function, resulting in the immunodeficiency associated with AIDS [61]. Various HIV-1 proteins were shown to interact with the host WTp53 during the process of infection, and thus to either attenuate or activate it, depending on infection stage. In early phase, HIV-1 proteins such as Nef and HIV-1 LTR were suggested to inactivate p53. The viral protein Nef, for example, interacts directly with p53 via its N terminus. This interaction results in the destabilization of p53, thereby decreasing p53 transcriptional activity and apoptosis [62]. Moreover, the upstream elements of the HIV-1 LTR, including the nuclear factor kappa B (NF-KB) binding sites, decrease the p53 inhibitory effects on viral transcription [63]. In later stages, other HIV-1 proteins were suggested to induce p53 activity, supporting HIV-1 prosperity. For example, Tat inhibits the host protein SIRT1, and by that removes SIRT1 inhibition on p53. This activates p53 to induce the expression of its target genes, *p21* and *BAX*, leading to host cells’ death [64]. Additionally, the viral protein, Vif, affects p53 stability by blocking the MDM2- p53 interactions. This mediates a G2 arrest, which positively supports HIV-1 replication [65]. Interestingly, positive regulatory loops are suggested. It was shown that overexpression of WTp53 decreases the activation level of the HIV-1 LTR via the viral protein, Vpr [66]. Additionally, Mukerjee et al. have shown that overexpression of WTp53 mediates inhibition of the transcriptional elongation HIV-1 LTR via phosphorylation of RNA polymerase II [67]. This HIV-1 LTR attenuation in turn might lead to further p53 activation.

### 3.5. The Human Herpes Simplex Virus 1

The human herpes simplex virus-1 (HSV-1) genome is a linear double-stranded DNA virus [68]. Accumulated data have shown that p53 plays a dual role in HSV-1 replication at different stages of its infection [69]. On one hand, p53 supports HSV-1 replication by inducing the expression of the viral protein ICP27 that is essential for the HSV-1 replication at early stages of the infection [69]. This positive p53 regulation was also corroborated in in vivo experiments, where higher HSV-1 replication and mortality rates were noticed in p53WT mice compared with their p53KO counterparts [70]. On the other hand, p53 mediates the degradation of the viral protein ICP0, which is also essential for both viral replication and a host immune response repression, and by that attenuates HSV-1 replication [69]. In addition, p53 responsive elements (RE) were shown to reside adjacent to different viral immediate-early (IE) genes, such as ICP4 and ICP8, which are critical for the HSV-1 life cycle [71]. By that, it is suggested that p53 inhibits the expression of these genes and HSV-1 replication, thus providing insight into the negative impact of p53 on the expression of vital HSV-1 functions and replication [72]. Interestingly, HSV-1 manages to antagonize this negative impact of p53 via the viral protein ICP22, which is able to bind p53 directly and abolish its function [69].

## 4. Targeting WTp53 as a Future Clinical Approach for Viral Infections

p53 regulation was found to play a crucial role in different infection stages of various viruses. Therefore, it is rational to believe that novel treatments, based on targeting and reactivating p53, may lead to beneficial therapeutic outcomes not only for cancer therapy but also for infectious diseases at large. Indeed, super p53 mice, harboring an extra copy of p53, showed better tumor resistance as well as antiviral activity [73]. Accordingly, some preliminary proof of concept experiments have shown that p53 may serve as a therapeutic target for viral infections. For example, p53 activation and induction of p53-dependent apoptosis may function as such antiviral therapy. Recently, a treatment for HPV‏ head and neck squamous cell carcinoma (HNSCC), which involves the use of 5-Azacytidine (5-aza), was suggested. It was found that by mediating DNA demethylation in host cells, 5-aza led to several outcomes; on the one hand, it reduced the expression of HPV genes and on the other hand, it caused p53 protein stabilization that in turn induced p53-dependent apoptosis [74]. Others have shown that treatment with anticancer chemotherapeutic agents, such as 5-fluorouracil (5-FU), can be used also against HIV-1 latently-infected T-cells, since 5-FU also stabilizes p53 leading to increased levels of p53-dependent apoptosis [75].

All in all, it seems that the incessant effort for developing drugs that will be able to target and activate WTp53, such as Nutlin, is crucial not only for cancer therapy but also to a greater extent for different antiviral infections.

## 5. Conclusions

**Figure 1:**
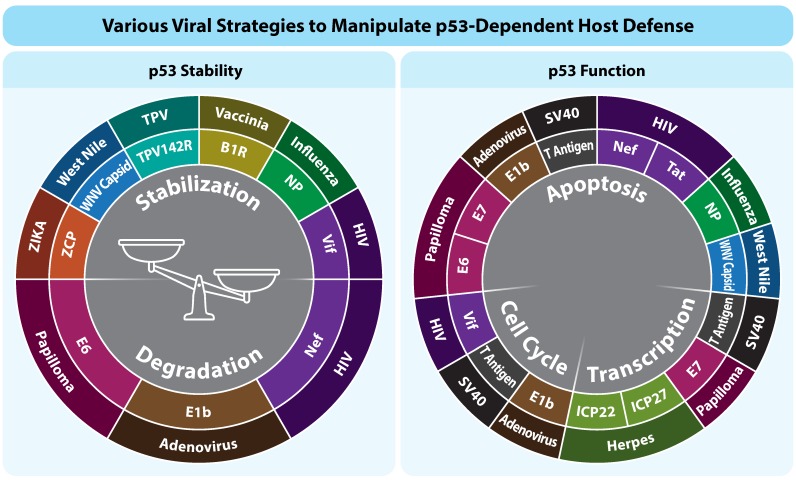
Various viral strategies to manipulate p53-depndent host defense.

Viruses are dependent on their cellular host for their prosperity, thus they have adopted different strategies to recruit the host cell machinery for their own needs. p53, being an important cellular transcription factor, orchestrates main cellular pathways which determine the cell fate. Therefore, it is not surprising that WTp53 is a prominent candidate for viral targets (Table 1). Viruses may target p53 in different modes. While some viruses need active p53, others dysregulate p53 in order to proliferate. Interestingly, some viruses act in a stage-specific dual manner regarding p53 regulation, activating at some stages of the viral cycle and inhibiting in others (Figure 1, Table 2). Despite the opposing viral strategies, each virus benefits according to its biological identity. Overall, tumor-promoting viruses reduce p53 activity or use p53 not in its “classical” functions. On the other hand, non-tumor-promoting viruses generally use p53 in its classical functions in order to proliferate and spread. WTp53 is known to participate in the innate immunity response to viral infection. p53 promotes and regulates the expression of the type I IFN response, as well as the induction and modulation of various cytokines and macrophage function [76,77]. Therefore, it is still an intriguing open question how viruses that activate the WTp53 overcome the normal classical function of WTp53 as a host defense player.

## Figures and Tables

**Table 1:** Various viral proteins and their interactions with p53.

Virus	Viral Protein	Interaction with p53/Influencing p53	Outcome	Ref.
SV40	T-antigen	Binds to p53	p53 bridges the interaction between p300/CBP and T-antigen Allows progression to S phase	[20,23]
Adenovirus	E1B	Binds to p53	Allows progression to S phase	[20]
Papilloma virus	1. E6 2. E7	1. Interacts with E3 ubiquitin ligase, E6AP 2. Connects directly with DREAM components p107 and p130	1. Degradation of p53 2. Disruption in p53 regulation	[28] [33,34]
Vaccinia virus	B1R kinase	Hyperphosphorylation of p53, significant reduction in the acetylation of p53 by p300	Increase in p53 ubiquitination and stability	[36,37]
Tanapoxvirus	TPV142R	Phosphorylation of p53	To be determined	[39]
ZIKA virus	C-terminus of the ZIKA capsid protein (ZCP)	Interacts with MDM2 and interferes with the formation of MDM2 and p53 complex	High levels of activated p53	[46]
West Nile virus	WNV capsid	Interferes with the formation of the HDM2 and p53 complex	Stabilization of p53 and the subsequent induction of its target apoptotic protein, Bax.	[48]
Influenza virus	viral nucleoprotein (NP)	1. Association of NP with p53 2. NP interacts with the host protein RING finger protein 43, a RING-type E3 ubiquitin ligase	1. Impairs the Mdm2-mediated p53 ubiquination and the interaction between p53 and Mdm2 2. Modulates p53 ubiquitination levels, leading to p53 stabilization and enhanced apoptosis	[54,55]
Human immuno-deficiency virus type 1	1. Nef 2. Tat 3. Vif	1. Direct interaction with p53 2. Inhibition of SIRT1 3. Blocks MDM2-mediated degradation and nuclear export of p53	1. Destabilization of p53 2. Activation of p53 pathway (p21 and Bax) 3. G2 arrest	[62] [64] [65]
Human herpes simplex virus-1	1. ICP22 2. ICP27	1. Interacts with p53 2. p53 induces its expression	1. Antagonizes the p53-dependent degradation of the viral protein ICP0. 2. HSV-1 replication	[69]

**Table 2:** Characterization of p53/virus interactions.

Viruses that Require Reduction/Inhibition of p53	Viruses that Require both Activation and Reduction of p53 during Infection, in a Stage-Dependent Manner	Viruses that Require Activation of p53
Adenovirus [9,20]	SV40 [24]	Zika [46]
Vaccinia [36]	Influenza A virus [56,57,58]	West Nile [48]
Tanapoxvirus [39]	HIV-1 [62]	
Human papillomavirus [28,33,34]	HSV-1 [70]	
